# Muscle Sympathetic Nerve Activity Is Associated with Liver Insulin Sensitivity in Obese Non-Diabetic Men

**DOI:** 10.3389/fphys.2017.00101

**Published:** 2017-02-28

**Authors:** Daniel L. T. Chen, Rachael Brown, Carsten Liess, Anne Poljak, Aimin Xu, Jialiang Zhang, Michael Trenell, Arthur Jenkins, Donald Chisholm, Dorit Samocha-Bonet, Vaughan G. Macefield, Jerry R. Greenfield

**Affiliations:** ^1^Diabetes and Metabolism Division, Garvan Institute of Medical ResearchSydney, NSW, Australia; ^2^School of Medicine, University of Western SydneySydney, NSW, Australia; ^3^Neuroscience Research AustraliaSydney, NSW, Australia; ^4^Philips HealthcareLuebeckertordamm, Hamburg, Germany; ^5^Bioanalytical Mass Spectrometry Facility, UNSW SydneySydney, NSW, Australia; ^6^School of Medical Sciences, UNSW SydneySydney, NSW, Australia; ^7^State Key Laboratory of Pharmaceutical Biotechnology, University of Hong KongHong Kong, Hong Kong; ^8^Movelab, Newcastle UniversityNewcastle, UK; ^9^School of Health Science, University of WollongongWollongong, NSW, Australia; ^10^Department of Endocrinology and Diabetes Center, St. Vincent's HospitalSydney, NSW, Australia

**Keywords:** obesity, insulin resistance, muscle sympathetic nervous activity, men, liver insulin sensitivity

## Abstract

**Introduction:** Muscle sympathetic nerve activity (MSNA) may play a role in insulin resistance in obesity. However, the direction and nature of the relationship between MSNA and insulin resistance in obesity remain unclear. We hypothesized that resting MSNA would correlate inversely with both muscle and liver insulin sensitivity and that it would be higher in insulin-resistant vs. insulin-sensitive subjects.

**Materials and methods:** Forty-five non-diabetic obese subjects were studied. As no significant relationships were found in women, the data presented in on 22 men aged 48 ± 12 years. Two-step (15 and 80 mU/m^2^/min) hyperinsulinaemic-euglycaemic clamps were performed using deuterated glucose to determine liver and muscle insulin sensitivity. Clinical and metabolic parameters were assessed. MSNA was measured via a microelectrode inserted percutaneously into the common peroneal nerve.

**Results:** MSNA burst frequency correlated inversely with liver insulin sensitivity (*r* = −0.53, *P* = 0.02) and positively with the hepatokines C-reactive protein (CRP) and fibroblast growth factor (FGF)-19 (*r* = 0.57, *P* = 0.006, and *r* = −0.47, *P* = 0.03, respectively). MSNA burst frequency was lower in Liver_sen_ compared to Liver_res_ (27 ± 5 vs. 38 ± 2 bursts per minute; *P* = 0.03). Muscle insulin sensitivity was unrelated to MSNA.

**Discussion:** Sympathetic neural activation is related to liver insulin sensitivity and circulating hepatokines CRP and FGF-19 in non-diabetic obese men. These results suggest a potential hepato-endocrine-autonomic axis. Future studies are needed to clarify the influence of MSNA on liver insulin sensitivity in men.

## Introduction

The sympathetic nervous system (SNS) plays an important role in homeostasis and metabolism, influencing resting metabolic rate, energy expenditure, and glucose and lipid metabolism (Astrup et al., 1995; Stob et al., 2007). Resting muscle sympathetic nerve activity (MSNA) regulates vasoconstriction, and is a major determinant in the control of blood pressure. MSNA has been shown to be higher in normotensive obese vs. lean individuals (Scherrer et al., 1994; Grassi et al., 1995). It has also been shown to decrease with weight loss (Straznicky et al., 2010).

Obesity is associated with insulin resistance and other metabolic complications, possibly contributed to by impaired sympathetic nerve activity. Hyperinsulinaemia is commonly observed in obesity; it is closely linked to peripheral and hepatic insulin resistance and has been shown to stimulate MSNA in humans (Landsberg, 1996; Greenfield and Campbell, 2008). Higher baseline MSNA and a blunted MSNA response to oral glucose is a characteristic of obese insulin-resistant individuals when defined by indirect oral glucose tolerance test (OGTT) indices (Straznicky et al., 2009). However, the nature and direction of the association between SNS activity and peripheral and hepatic insulin resistance remain unclear.

Muscle sympathetic nerve activity, measured directly using a microelectrode inserted percutaneously into a peripheral nerve (microneurography), is a reliable and reproducible assessment of sympathetic nerve activity (Grassi and Esler, 1999). Although MSNA accounts for only about 20% of whole body release of norepinephrine to plasma, it has been shown to be closely related to cerebral, cardiac, and renal noradrenaline spillover (Wallin et al., 1992, 1996; Lambert et al., 1997; Straznicky et al., 2008). There is a gender difference in MSNA among obese individuals (Brooks et al., 2015). MSNA has been shown to correlate with Body Mass Index (BMI), visceral adiposity, and waist circumference in obese men (Lambert et al., 2007), but not in premenopausal or postmenopausal women (Brooks et al., 2015). The reasons for this gender specificity are not completely understood, but it is proposed to relate to differences in adipose tissue disposition, inflammatory profiles, and pressor and depressor components of the renin aldosterone angiotensin system (Fischer et al., 2002; Brooks et al., 2015). Estrogen may play a role in resisting obesity-induced hypertension and MSNA elevation by suppressing inflammatory cytokines and reducing production and actions of pressor renin-aldosterone system components (Brooks et al., 2015), supported by differences observed in MSNA correlations between pre and postmenopausal women (Brooks et al., 2015).

We evaluated resting MSNA in 45 non-diabetic obese subjects who had undergone a two-step hyperinsulinaemic-euglycaemic clamp with deuterated glucose to assess liver and muscle insulin sensitivity (Chen et al., 2015). We hypothesized that resting MSNA would be correlated with both muscle and liver insulin sensitivity and that in subjects matched for adiposity, MSNA would be higher in liver and muscle insulin-resistant subjects compared to their insulin-sensitive counterparts.

## Materials and methods

This is a subcomponent of a parent study that has been reported elsewhere (Chen et al., 2015). One hundred and eighty-four subjects aged 21–69 years were recruited from the general population by advertisements in local newspapers. The study was approved by St Vincent's Hospital Human Research Ethics Committee, Sydney. Subjects provided informed written consent prior to commencement of the study, which was conducted under the guidelines of the Declaration of Helsinki. Exclusion criteria were known renal, cardiac or liver disease, or active cancer and diabetes, >5% change in body weight in the preceding 3 months, medications that affect glucose metabolism (including diabetes medications, anti-psychotics and steroids) and >20 g of alcohol; based on these criteria 104 potential participants screened over the phone were excluded from the study.

The remaining 80 subjects underwent standard OGTT. Diabetes was defined according to the American Diabetes Association criteria (American Diabetes Association, 2014). Fourteen subjects were excluded due to difficult venous access or loss of interest. Of the 66 subjects remaining, all agreed to undergo MSNA studies; 45 (22 males) were successfully studied. Twenty one subjects (7 males) did not have successful MSNA recording due to technical issues or poor recording quality. As no relationships were found between MSNA indices and insulin resistance in females, data on males only are presented. No subject was treated with beta-blockers. Clamp studies were performed at the Clinical Research Facility at the Garvan Institute of Medical Research, Sydney as previously described (Milner et al., 2010). Recording of MSNA was performed at Neuroscience Research Australia (NeuRA), Sydney and magnetic resonance imaging (MRI) and dual-energy X-ray absorptiometry (DXA) at St Vincent's Hospital, Sydney.

### Definition of insulin sensitivity in muscle and liver

All subjects underwent two-step (insulin infusion rates 15 and 80 mU/m^2^/min) hyperinsulinaemic-euglycaemic clamps with deuterated glucose (6,6-^2^H_2_, Cambridge isotope, Andover) to assess muscle and liver insulin sensitivity as described previously (Milner et al., 2010; Petersons et al., 2013; Chen et al., 2015). The percentage of endogenous glucose production (EGP) suppression with low-dose insulin was used to define liver insulin sensitivity.

Liver insulin-sensitive men (Liver_sen_) were defined as the top tertile of EGP suppression and liver insulin-resistant men (Liver_res_) as the bottom two tertiles, as previously described (Chen et al., 2015). The cut-off was based on an average prevalence of 30% insulin-sensitive obesity amongst the obese population in large cohort studies (Pataky et al., 2010).

Muscle insulin sensitivity was inferred from the glucose infusion rate (GIR) during the high-dose insulin clamp (GIR_HI_) adjusted for fat-free mass (FFM). Study participants were assigned to the muscle insulin-sensitive (Muscle_sen_) group if GIR_HI_ was in the top tertile of the cohort and to the muscle insulin-resistant (Muscle_res_) group if in the bottom two tertiles. Our Muscle_sen_ group had comparable GIR_HI_ to that measured in a group of lean healthy individuals previously studied in our Clinical Research Facility using a similar protocol (Tonks et al., 2013). As EGP was fully suppressed during the high-dose insulin infusion, GIR_HI_ reflects peripheral (mainly muscle) insulin sensitivity.

### Anthropometry and body composition

BMI was defined as weight in kilograms divided by height in meters squared. Waist circumference was measured as the widest circumference between the lower end of the ribs and the anterior superior iliac spines. Hip circumference was measured at the widest circumference between the greater trochanters and the anterior superior iliac spines. Total body fat mass, FFM and central abdominal fat were measured by DXA (Lunar Prodigy, GE-Lunar, Madison, WI, USA; S/ware 12.20). In some larger subjects, analysis was performed by aligning one side of the body on the scanner, and doubling the data to achieve overall body composition, as previously described (Chen et al., 2015).

### Magnetic resonance imaging

MRI (3.0 T Philips Achieva, Philips Medical Systems, The Netherlands) images were acquired using the mDIXON technique to evaluate the amount of liver and subcutaneous fat. The amount of visceral adipose tissue was measured in five slices at the L4/L5 intervertebral disc level and quantified using Image J software 1.46r (NIH, USA). As described previously (Milner et al., 2010; Chen et al., 2015), visceral fat was calculated as the difference between total fat and subcutaneous fat. One subject was over the weight limit of the MRI machine and so quantitative fat data were not available for this subject.

### Muscle sympathetic nerve activity and blood pressure measurement

Measurements of resting MSNA were performed in a quiet room at 22°C in the seated position on a separate day to the clamp study. Subjects voided before commencement. MSNA was performed in a resting state with one-third of subjects studied in the afternoon due to attendance at the Garvan Institute in the morning. Subjects refrained from caffeine for 4 h prior to recording.

Subjects lay in a chair with their backs at 45° angle and legs supported horizontally. Spontaneous resting MSNA was recorded by inserting an insulated tungsten microelectrode (Frederick Haer and Co., Bowdoinham, ME, USA) percutaneously at the level of the fibular head into the left common peroneal nerve and advanced toward a muscle fascicle of the nerve while delivering weak electrical impulses (0.01–1 mA, 0.2 ms, 1 Hz). An uninsulated reference electrode was inserted subdermally 2–3 cm from the recording electrode and a surface Ag–AgCl electrode applied to the leg served as the ground electrode. A muscle fascicle was defined as such if intraneural stimulation evoked muscle twitches of the ankle, toe dorsiflexors, or foot everter muscles with no radiating paraesthesia. Once a muscle fascicle had been entered, neural activity was amplified (band-pass 0.3–5 kHz, gain 2 × 10^4^) using an isolated amplifier and headstage (NeuroAmpEx, AD Instruments, Sydney, Australia). The position of the electrode was then adjusted manually until spontaneous bursts of MSNA were identified. Once an acceptable nerve-recording site was obtained with both visual and acoustic identification of spontaneous sympathetic bursts, resting measurements were recorded and stored on computer (10 kHz sampling) using a computer based data acquisition and analysis system (PowerLab 16SP hardware and LabChart 7 software; AD Instruments). Continuous non-invasive blood pressure was recorded using radial artery tonometry (CBM-7000, Colin Corp., Japan). The ECG (0.3 Hz–1 kHz) was recorded with surface electrodes on the chest and sampled at 2 kHz and respiration (DC-100 Hz).

MSNA burst amplitudes were measured from the RMS-processed signal (200 ms moving average) using the Peak Parameters feature of LabChart7 (AD Instruments, Sydney, Australia). The entire process has been described previously (Macefield, 2013). MSNA was manually analyzed and expressed as burst frequency (bursts per minute) and burst incidence (bursts per 100 heart beats), averaged over 15 min. There were no significant differences in MSNA -measured variables between subjects in whom MSNA was recorded in the morning or the afternoon (morning *n* = 15; afternoon *n* = 7; independent *t*-test; *P* > 0.47).

### Laboratory analyses

Whole blood glucose was measured using the YSI 2300 STAT analyzer (Yellow Springs Ohio). Serum insulin and C-peptide concentrations were measured by radioimmunoassay (Millipore, St Charles, USA). Plasma lipid profiles were determined by an automated analyzer (Roche, IN, USA). Serum non-esterified fatty acids (NEFA) were analyzed by an enzymatic colorimetric assay (Wako, Osaka, Japan). Serum high sensitivity C-reactive protein (hsCRP), fibroblast growth factors FGF-19 and FGF-21, total adiponectin, fatty acid-binding protein 4 (FABP4), lipocalin 2 and retinol-binding protein 4 (RBP4) were analyzed by ELISA (Antibody and Immunoassay Services at the University of Hong Kong; Chen et al., 2015). The intra- and inter-assay CVs have been reported previously (Chen et al., 2015).

### Statistical analysis

Data were expressed as means ± *SEM* unless otherwise specified. Non-normally distributed data (serum triglycerides, insulin, NEFA, hsCRP, FGF-19, and FGF-21) were transformed logarithmically prior to statistical analysis. Student's *t*-test was used to evaluate significant differences between the different phenotypes. Correlations were performed using Pearson's correlation analysis. Chi-square and Fisher's exact test were used to detect differences in timing of MSNA measurement in Liver_sen_ and Liver_res_ groups. *P* < 0.05 was considered statistically significant. Statistical analysis was carried out using SPSS version 21 (Chicago, IL, USA).

## Results

Baseline characteristics are presented in Table 1. Mean age was 48 ± 2 years and BMI was 34.7 ± 0.7 kg/m^2^ (range 30.9–46.7 kg/m^2^). Mean systolic and diastolic blood pressure were 127 ± 14 and 83 ± 11 mmHg, respectively. Mean MSNA-derived measures were 56 ± 17 bursts per minutes and 34 ± 11 burst per 100 heart beats. Five male subjects reported taking anti-hypertensive medications. There were no significant differences in MSNA burst frequency (*P* = 0.53) and MSNA burst incidence (*P* = 0.30) between men taking and not taking anti-hypertensive medications. Furthermore, no significant differences in average HbA1c between men treated and men not treated with anti-hypertensive medications was found (*P* = 0.86).

**Table 1:** **Clinical characteristics**.

	**Characteristics (***n*** = 22)**	
Age	Age (years)	48 ± 12
Adiposity	BMI (kg/m^2^)	34.7 ± 3.3
	Waist circumference (cm)	114 ± 9
	Whole body fat (kg)	40 ± 7
	Fat-free mass (kg)	64 ± 6
	Central abdominal fat (kg)	3.7 ± 0.6
	Subcutaneous fat (cm^2^)	441 ± 117
	Liver fat (%)	16 ± 15
Blood pressure	SBP (mmHg)^**^	127 ± 14
	DBP (mmHg)^**^	83 ± 11
MSNA-derived measures	Burst incidence (bursts/100 beats)	56 ± 17
	Burst frequency (bursts/min)	34 ± 11
	Heart rate (BPM)	66 ± 7
Fasting lipid profile	Total cholesterol (mmol/L)^∧^	5.0 ± 0.9
	LDL cholesterol (mmol/L)^∧^	3.3 ± 0.8
	HDL cholesterol (mmol/L)^∧^	1.1 ± 0.2
	Triglycerides (mmol/L)^∧^^Φ^	1.1 (0.8–1.5)
	NEFA (mmol/L)^Φ^	0.27 (0.25–0.32)
Fasting glycaemic parameters	HbA1c (%)	5.5 ± 0.3
	Fasting glucose (mmol/L)	4.8 ± 0.4
	Fasting insulin (mU/L)^Φ^	18 (12–26)
Serum fasting cytokines	hsCRP (mg/L)^Φ^	2.2 (1.4–4.9)
	FGF 19 (ng/L)^Φ^	97 (56–152)
	FGF 21(ng/L)^Φ^	49 (16–159)
	FABP 4 (μg/L)	42 ± 17
	Lipocalin 2 (μg/L)	44 ± 14
	RBP 4 (mg/L)	12 ± 2
	Total adiponectin (mg/L)	10 ± 4

*Data are means ±SD*.

Φ Data are median (interquartile range) for non-normally distributed data

** *Subjects treated with anti-hypertensive medications excluded from the analysis (included: n = 18)*.

∧ *Subjects treated with lipid lowering medications excluded from the analysis (included: n = 17)*.

*SBP, systolic blood pressure; DBP, diastolic blood pressure; BPM, beat per minute; LDL, low density lipoprotein; HDL, high density lipoprotein; hsCRP, highly sensitive C-reactive protein; FGF, Fibroblast growth factor; FABP4, Fatty acid binding protein 4; RBP4, Retinol binding protein 4*.

### Linear regression analyses

**Figure 1:**
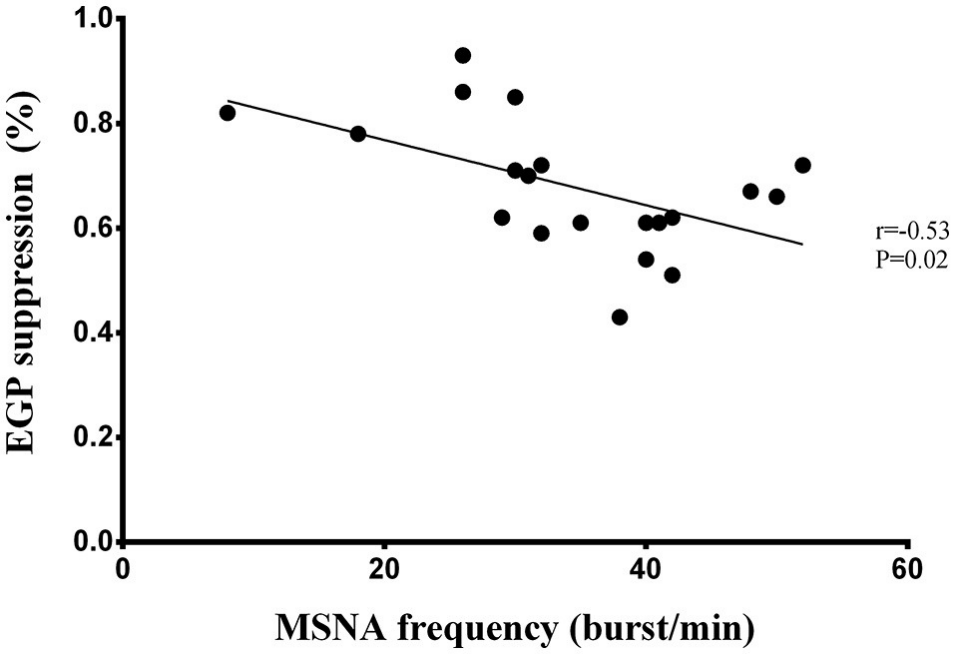
**Correlation between liver insulin sensitivity and MSNA burst frequency in men**.

Liver insulin sensitivity (expressed as EGP suppression) correlated inversely with MSNA burst frequency (*r* = −0.53, *P* = 0.02; Figure 1), but the inverse relationship with MSNA burst incidence failed to reach statistical significance (*r* = −0.36, *P* = 0.12). Fasting insulin tended to correlate with MSNA burst frequency (*r* = 0.42, *P* = 0.05), while muscle insulin sensitivity was not related to either MSNA burst frequency (*P* = 0.09) or burst incidence (*P* = 0.4).

**Figure 2:**
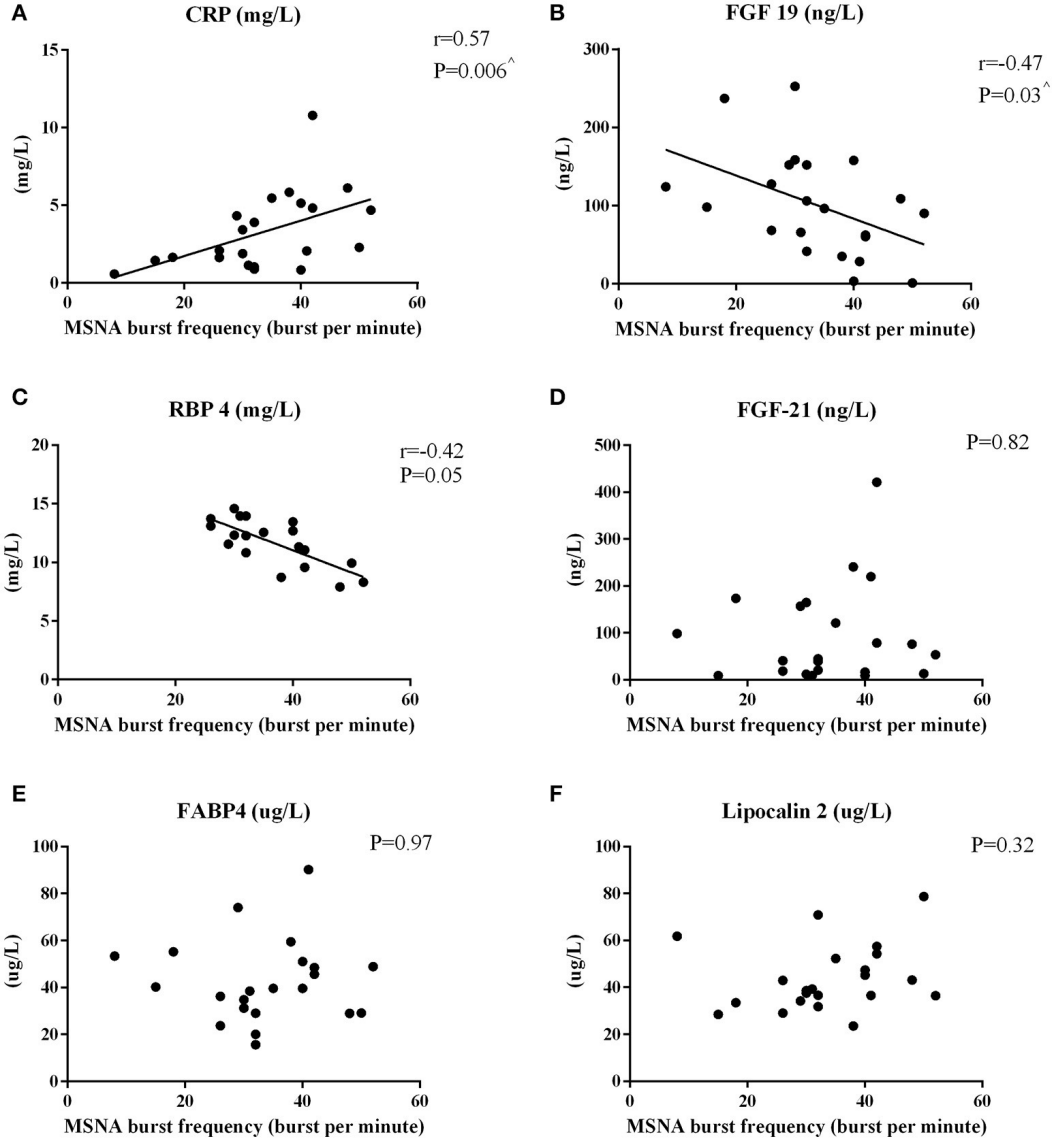
**Pearson's Correlations between MSNA burst frequency and circulating hepatokines in men**. The correlations between MSNA burst frequency and hsCRP **(A)**, FGF-19 **(B)**, RBP4 **(C)**, FGF-21 **(D)**, FABP4 **(E)**, and lipocalin-2 **(F)** are reported. ^∧^*P* < 0.05 after adjusting for total body fat.

MSNA burst frequency and incidence correlated positively with hsCRP (Figure 2A and *r* = 0.45, *P* = 0.04, respectively). FGF-19 correlated (Figure 2B) and RBP4 tended to correlate inversely with MSNA frequency (*P* = 0.05, Figure 2C). Factors unrelated to MSNA-derived variables were FGF-21 (Figure 2D), FABP4 (Figure 2E), lipocalin 2 (Figure 2F), and total adiponectin (*P* > 0.16).

Age correlated positively with MSNA burst incidence (*r* = 0.61, *P* = 0.002), but the relationship to burst frequency failed to reach statistical significance (*r* = 0.37, *P* = 0.09). Systolic blood pressure, diastolic blood pressure, subcutaneous and liver fat, serum triglycerides and HbA1c were not related to either MSNA burst incidence or frequency (*P* > 0.09).

### Stratification by liver insulin sensitivity

Given the significant inverse correlation between MSNA and liver insulin sensitivity, we stratified the cohort according to liver insulin sensitivity. The cohort was stratified based on EGP suppression level to Liver_sen_ and Liver_res_ (top tertile vs. bottom two tertiles, 72% EGP suppression as cut-off value).

**Figure 3:**
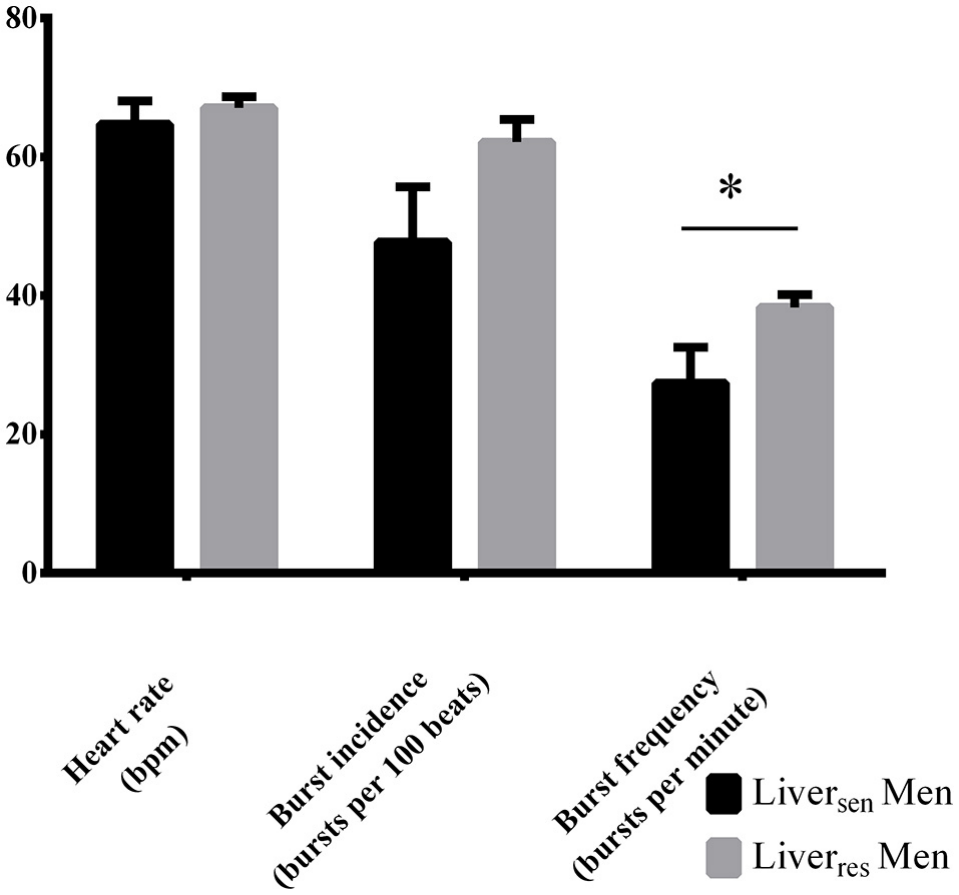
**Heart rate, burst incidence and burst frequency measured by MSNA in obese men stratified by liver insulin sensitivity**. Differences between groups were analyzed by Student *T*-test, ^*^P < 0.05.

Liver_sen_ men had lower HbA1c (5.2 ± 0.1 vs. 5.6 ± 0.1%, *P* = 0.01), fasting insulin (14 [11–22] vs. 25 [17–41] mU/L, *P* = 0.03) and hsCRP (1.7 [1.0–2.1] vs. 4.3 [2.2–5.7] mg/L, *P* = 0.03) than Liver_*res*_ men, but had similar age (*P* = 0.14), BMI (*P* = 0.79), central abdominal fat (*P* = 0.58), and systolic (*P* = 0.10) and diastolic (*P* = 0.66) blood pressure. MSNA burst frequency was significantly lower in Liver_sen_ men compared with Liver_res_ men (27 ± 5 vs. 38 ± 2; *P* = 0.03, Figure 3). MSNA burst incidence tended to be lower in Liver_sen_ men (Figure 3, *P* = 0.06).

## Discussion

To our knowledge, this is the first study to directly assess liver insulin sensitivity and MSNA in obese non-diabetic subjects. Basal SNS activity measured by MSNA was closely associated with liver insulin sensitivity in men with obesity. The metabolic profile of the insulin-sensitive men with obesity in the present study was consistent with previous reports, including lower glycaemia, fasting insulin, and CRP (Samocha-Bonet et al., 2012). The present study highlights the importance of the cytokines CRP and FGF-19 in their correlations with MSNA in obese men. These findings suggest involvement of a potential hepato-endocrine-sympathetic axis.

We have shown that MSNA was lower in liver insulin-sensitive obese men. Previous studies that used hyperinsulinaemic-euglycaemic clamps and microneurography (Straznicky et al., 2012; Curry et al., 2014) did not report any gender specific differences in the relationship between MSNA and whole body insulin sensitivity. Nevertheless, these studies included younger (18–35 years) non-obese (BMI < 28 kg/m^2^) individuals (Curry et al., 2014) or pre-diabetic and diabetic individuals (Straznicky et al., 2012), making comparisons with our study difficult. MSNA has been shown to be regulated differently in men and women (Scherrer et al., 1994; Lambert et al., 2007).

Previous studies have reported that resting MSNA was higher in obese insulin-resistant subjects compared to their insulin-sensitive counterparts when defined by OGTT (Straznicky et al., 2009). The OGTT-derived Matsuda and Defronzo index of insulin sensitivity measures a composite of hepatic and muscle insulin action (Matsuda and DeFronzo, 1999) and our findings refine these and suggest that the relationship between insulin sensitivity and resting MSNA reported previously is predominantly contributed by the liver.

Serum FGF-19 correlated inversely with resting MSNA and was higher in insulin-sensitive compared with insulin-resistant men. FGF-19 has been shown to coordinate bile acid and glucose metabolism (Kharitonenkov et al., 2005). Liver is the main target organ of FGF-19 action, where FGF-19 binds to the FGFR 4/β-klotho complex to reduce gluconeogenesis and triglycerides in the liver (Cicione et al., 2012). FGF-19 functions in a coordinated temporal fashion with insulin to inhibit gluconeogenesis and promote glycogen synthesis after a meal (Cicione et al., 2012). FGF-19 and insulin may exert differential actions when they are involved with the SNS, as FGF-19 correlated inversely with MSNA, compared with a positive correlation between fasting insulin and MSNA. We also demonstrated a positive correlation between hsCRP and resting MSNA. While these associations cannot determine cause and effect, they may suggest the existence of a hepato-endocrine-autonomic axis, where liver-related cytokines play a role in regulating sympathetic nerve activity or vice versa. Further, clamp studies using deuterated glucose tracers to measure hepatic insulin sensitivity and noradrenaline spillover to measure sympathetic nerve activity on larger cohorts are needed to clarify the nature of the associations between hepatic insulin sensitivity, cytokines and sympathetic nerve activity.

## Limitations

Our study has some limitations. First, MSNA was not uniformly measured at the same time of the day. Nevertheless, MSNA measurements (MSNA burst frequency/incidence) performed at different times (morning versus afternoon) were not different. There were also no significant differences between the proportions of men in the higher MSNA measurement group (stratified by top tertile of MSNA burst incidence/frequency) performed in the morning or afternoon (chi-square *p* > 0.64). This finding has been supported by previous studies where MSNA, measured in the non-fasting state, did not differ between the morning and afternoon (Middlekauff and Sontz, 1995; Hissen et al., 2015). Second, MSNA measured in the peroneal nerve does not directly assess hepatic sympathetic nerve activity. Third, we recognize that this is an observational study that cannot infer causality. Finally, dissociation between regional and systemic sympathetic nerve activity has been described previously and regional noradrenaline spillover measurement may have more precisely assessed regional sympathetic nerve activity (Grassi and Esler, 1999).

In summary, basal sympathetic outflow to the muscle vascular bed is lower in insulin-sensitive compared to insulin-resistant men. Resting MSNA is inversely related to liver insulin sensitivity and may be mediated by liver-related factors in non-diabetic obese men. Further studies are needed to examine this putative hepato-endocrine-autonomic axis.

## Ethics statement

This study was carried out in accordance with the recommendations of St Vincent's Hospital Human Research Ethics Committee, Sydney. All subjects gave written informed consent in accordance with the Declaration of Helsinki. The protocol was approved by the St Vincent's Hospital Human Research Ethics Committee, Sydney.

## Author contributions

DLC, RB, DC, DS-B, VM, and JG designed the studies. DLC and RB performed the clinical studies. DLC, RB, DS-B, AP, AX, and JZ performed laboratory analysis of the data collected. DLC, MT, and CL performed and assisted in measuring visceral and subcutaneous adipose tissue. DLC, RB, DC, DS-B, VM, AJ, and JG interpreted the data. DLC wrote the manuscript. All authors edited and approved the final version of the manuscript. JG, VM, and DS-B are the guarantors of this work, have full access to all the data, and take full responsibility for the integrity and the accuracy of data analysis.

## Funding

The study was funded by the National Health and Medical Research Council (NHMRC) and in part, by St. Vincent's Clinic Foundation, Australia. DLC is funded by an Australian Postgraduate Awards (APA) Scholarship. JG is the recipient of the Don Chisholm Fellowship (funds from Garvan Research Foundation, including support from GlaxoSmithKline, Australia, Diabetes Australia Research Trust, the Commonwealth Department of Health and Ageing). No funding bodies had any role in the study design, data collection and analysis, decision to publish or preparation of the manuscript.

### Conflict of interest statement

The authors declare that the research was conducted in the absence of any commercial or financial relationships that could be construed as a potential conflict of interest.
